# Attosecond field emission

**DOI:** 10.1038/s41586-022-05577-1

**Published:** 2023-01-25

**Authors:** H. Y. Kim, M. Garg, S. Mandal, L. Seiffert, T. Fennel, E. Goulielmakis

**Affiliations:** 1 Institut für Physik, Universität Rostock, Rostock, Germany; 2 Max Planck Institute for Solid State Research, Stuttgart, Germany

**Keywords:** Optical physics, Nanoscience and technology

## Abstract

Field emission of electrons underlies great advances in science and technology, ranging from signal processing at ever higher frequencies^1^ to imaging of the atomic-scale structure of matter^2^ with picometre resolution. The advancing of electron microscopy techniques to enable the complete visualization of matter on the native spatial (picometre) and temporal (attosecond) scales of electron dynamics calls for techniques that can confine and examine the field emission on sub-femtosecond time intervals. Intense laser pulses have paved the way to this end^3,4^ by demonstrating femtosecond confinement^5,6^ and sub-optical cycle control^7,8^ of the optical field emission^9^ from nanostructured metals. Yet the measurement of attosecond electron pulses has remained elusive. We used intense, sub-cycle light transients to induce optical field emission of electron pulses from tungsten nanotips and a weak replica of the same transient to directly investigate the emission dynamics in real time. Access to the temporal properties of the electron pulses rescattering off the tip surface, including the duration *τ* = (53 as ± 5 as) and chirp, and the direct exploration of nanoscale near fields open new prospects for research and applications at the interface of attosecond physics and nano-optics.

## Abstract (web summary)

By using sub-cycle light transients to induce optical field emission of electron pulses from tungsten nanotips and a weak replica of the same transient, investigation of attosecond emission dynamics in real time is demonstrated.

## Main

**Fig. 1:**
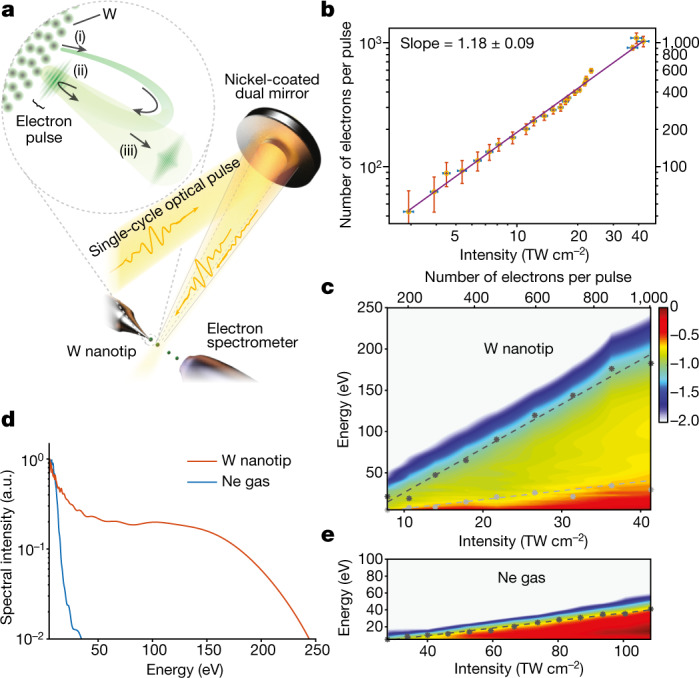
Optical field emission by intense, sub-cycle optical transients. **a**, Simplified schematic of the experimental setup. A sub-cycle pulse (orange curve) is spatially separated and focused by a dual concave Ni mirror module. A time delay between the pulses reflected by the inner and outer mirrors is introduced by a piezo stage. Tungsten nanotips (apex radius around 35 nm) or a gas jet of neon atoms can be positioned in the laser focus. Emitted electron spectra are recorded by a time-of-flight spectrometer (acceptance angle about 6°) placed roughly 3 mm downstream of the electron source and aligned along the laser polarization axis. The inset shows that electrons marked by the green shaded curve are (i) set free and accelerated by the intense laser field to form an electron pulse, which, on recollision with the nanotip surface, (ii) can investigate both dynamics as well as structure. On backscattering off the tip surface, (iii) the electron pulse is further accelerated by the laser to escape the interaction area. **b**, Total electron yield per pulse as a function of increasing peak intensity of the driving laser pulses (yellow points) and its linear fitting (purple line) on a logarithmic scale. **c**, Electron spectra from the tungsten nanotip (false colour in logarithmic scale) versus peak intensity. Stars and dots denote the cutoff energies. Black and grey dashed lines show the linear fitting of the cutoff energy versus incident peak intensity of the laser pulse. **d**, Optical emission electron spectra from the tungsten nanotip (red curve) and neon atoms (blue curve) for nearly identical peak intensity (about 40 TW cm^−2^). **e**, Same as in **c** but for neon atoms. Source data

The interaction of atoms and molecules with intense laser fields gives rise to attosecond electron pulses^10^ that can study the structure and dynamics of these systems on recollision with their parent ion^11^. Attosecond techniques^12^ can now gain access to the temporal profile of the recolliding electron pulses and concomitant structural dynamics^13,14^ in their parent ions by measuring the transient properties of high harmonics^15^ emitted during the interaction. Studies of the interaction of intense laser fields with nanostructured metals over the past two decades have suggested that the semiclassical concepts^3–9,16,17^ developed earlier to describe electron dynamics in atoms can afford a central role in the understanding of the optical field electron emission. In analogy to atoms, electrons set free from the apex of a nanotip at the field crest of an intense laser pulse should also form ultrashort electron pulses (Fig. 1a, inset), which—on recollision with the tip surface about three-quarters of the laser period (*T* ≈ 2 fs) later—could examine both dynamics and structure. Owing to the ultrashort time interval between emission and recollision events, and in contrast to other emerging electron-pulse technologies^18–21^, the electron-pulse wave packet shall undergo a negligible temporal spread, allowing its confinement to sub-cycle timescales.

However the real-time tracking of electron pulses generated in the optical field emission has remained challenging. Although ordinary attosecond streaking techniques can be used to map the temporal structure of the extreme ultraviolet (EUV) electron emission from metal surfaces^22^ and nanotips^23^, they cannot directly investigate electron pulses emerging in the optical field emission. Furthermore, the absence of high harmonic emission from laser-driven nanotips constrains the applicability of in situ attosecond techniques^24–27^ for examining the structure of the electron pulses in these systems.

Harnessing electron pulses emerging in the optical field emission for realizing new spectroscopies that combine attosecond temporal and nanometre spatial resolution calls for essential advancements on both their generation and their measurement methodologies. On the generation side, the driving laser pulses should be both short and intense so as to confine the tunnelling of electrons into a sub-femtosecond window (<1 fs), as well as to impart the recolliding electron pulses with a de Broglie wavelength (<2.74 Å, >20 eV) that allows atomic-scale investigation of the parent surface^28^. On the measurement side, in situ attosecond metrologies^24–27^ shall be extended to incorporate temporal gating of the optical field emission without relying on the concomitant high harmonic radiation. Measurements of this kind have so far permitted access to the driving-field waveform of light waves by tracking the spectrally integrated currents induced in the bulk of solids^29,30^ or the cutoff energy variation of rescattered electrons in atoms^31^ and nanotips^32^, but a direct time-resolved measurement of attosecond electron pulses in the optical field emission has remained beyond reach.

**Extended Data Fig. 1:**
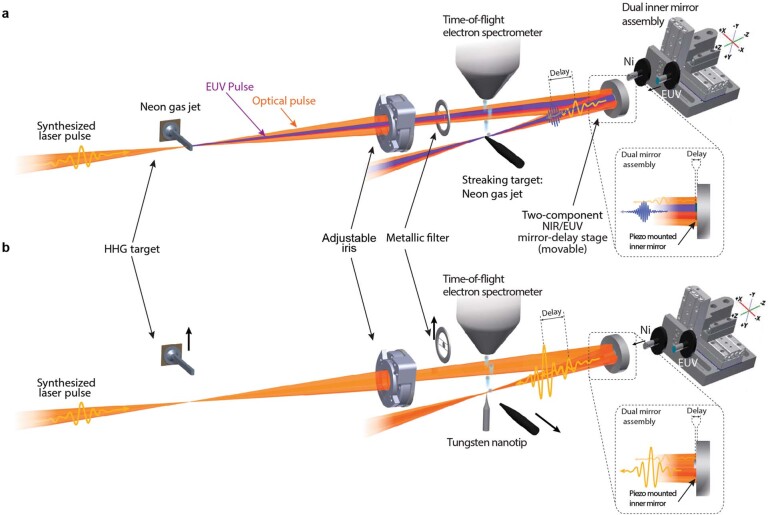
Experimental setup. EUV (**a**) and HAS (**b**) configurations. The schematics of the adjustable iris and the dual inner mirror assembly are reproduced with permission by SmarAct GmbH.

Guided by the above provisions, we studied the field emission in tungsten nanotips (work function *ϕ* ≈ 4.5–5.0 eV) using intense (around 10^13^ W cm^−2^), sub-cycle (about 1.9 fs) optical transients (centroid energy around 1.8 eV) generated in a light-field synthesizer^33,34^. The experiments were performed in a multifunctional experimental setup (see Methods and Extended Data Fig. 1) that combines photoemission spectroscopy of atoms and solids, optical pump–probe measurement methodologies and EUV attosecond streaking^35^ for the sampling of the driving-field waveforms.

**Extended Data Fig. 2:**
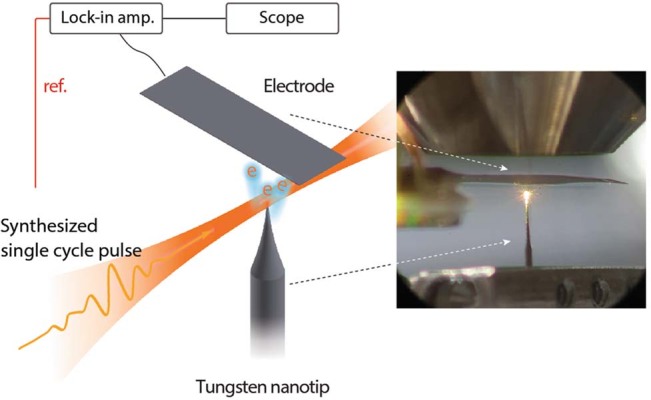
Module for the measurement of the absolute electron yield. Electrons emitted from a tungsten nanotip exposed to single-cycle pulses were captured by a thin electrode placed about 2 mm above the tip. The induced voltage on the electrode was recorded by a lock-in amplifier.

We began our experiments by analysing the nonlinearity of the electron yield to the driving-field intensity. A plate detector (see Extended Data Fig. 2) recorded the total number of emitted electrons from the electrically grounded nanotip (yellow points, Fig. 1b) for a range of peak intensities of the impinging laser transient. For the highest peak intensity of around 42 TW cm^−2^, we recorded up to about 1,000 electrons per pulse. Evaluation of the slope of the total electron yield versus peak intensity (purple line in Fig. 1b) showed an emission nonlinearity of about 1.18 ± 0.09, which is well below the multiphoton threshold *ϕ*/*ħω*_L_ ≈ 3 and supports the notion that field-driven electron tunnelling dominates the ionization of tungsten over the entire range of the studied intensities.

Next we conducted a spectral-domain study of the emission under precisely characterized driving fields and interrogated compatibility of our findings with the predictions of semiclassical, single-electron models. Given the multielectron nature of the emission (Fig. 1b), this step is essential for applying semiclassical single-electron concepts later in this work to scrutinize the temporal structure of the electron emission. As space–charge interactions can substantially distort the emission spectra, in comparison with semiclassical predictions, a detailed interrogation of the cutoff energies of the emission can shed light onto potential multielectron contributions.

Electron spectra recorded as a function of the peak intensity of the driving pulse (Fig. 1c) showed the formation of two well-discernible cutoffs (black points, grey points and false-colour plot in Fig. 1c, respectively), whose energies scale linearly with peak intensity. Evaluation of the corresponding slopes for high and low cutoff energies (*E*_c_) by linear fitting (black and grey dashed lines in Fig. 1c) of the experimental data yielded ${s}_{\text{W},\text{high}}^{(\exp )}={\rm{d}}{E}_{{\rm{c}}}/{\rm{d}}{U}_{{\rm{p}}}=118\pm 5.1$ and ${s}_{\text{W},\text{low}}^{(\exp )}={\rm{d}}{E}_{{\rm{c}}}/{\rm{d}}{U}_{{\rm{p}}}=24.1\pm 1.35$, respectively. Here *U*_p_ is the ponderomotive energy. By taking the ratio between high and low cutoff energy slopes (about 4.91 ± 0.35), we find that it is compatible with that anticipated for backscattered and direct electrons (10*U*_p_/2*U*_p_ ≈ 5) in the single-electron approximation^36^.

An experimental assessment of the near-field enhancement in the vicinity of the nanotip and its comparison with the theoretical predictions could further attest to the validity of single-electron semiclassical pictures to describe emission under our experimental conditions. To this end, we compared the emission spectra of electrons from the nanotip with those in a low-density neon gas. Figure 1d contrasts electron spectra recorded from W nanotip (red curve) and neon atoms (blue curve) under identical field waveforms. Figure 1e shows electron spectra emanating from Ne over a broad range of driving-pulse intensities. A linear fitting (Fig. 1e, dashed line) of the cutoff energy (Fig. 1e, points) of the neon spectra versus intensity yielded a slope ${s}_{{\rm{Ne}}}^{(\exp )}=9.88\pm 0.34$ that agrees well with the semiclassical predictions under our driving waveforms (*s*_Ne_ = 10.8) (see Methods). The field-enhancement factor in the vicinity of the tungsten nanotip, which is now evaluated as $f=\sqrt{\frac{{s}_{{\rm{W}},{\rm{high}}}^{(\exp )}}{{s}_{{\rm{Ne}}}^{(\exp )}}}=3.46\pm 0.10$, shows a fair agreement with the theoretical prediction, *f*_th_ ≈ 3.8 (Methods). This comparison further attests to the compatibility of the emission processes from the nanotip with single-electron, semiclassical concepts and suggests that multielectron charge interactions remain negligible.

Detailed semiclassical simulations based on the experimentally derived quantities (see Methods) further support the above perspective. The simulations accurately reproduce the experimental spectra (Extended Data Fig. 4) and the association of high and low cutoff energies of the emission to backscattered and direct electrons, respectively. The inclusion of multielectron interactions (Extended Data Fig. 6) showed the presence of weak-only effects of space–charge distortions on the emitted spectra and the spectral phase of the electron emission further corroborating the appropriateness of single-electron models to describe the emission process.

The absence of discernible cutoffs at intermediate energies (60–120 eV) in both experiments (Fig. 1c) and simulations (Extended Data Figs. 4 and 6) supports the notion that, under sub-cycle driving, the high-energy part of the emitted spectrum is associated with the recollision of a single electron pulse at the tip surface. A weak, low-energy backscattered emission indicated by the simulations (Extended Data Fig. 4a) is not directly resolved in the experiments (Fig. 1c). Yet the presence of such emission channels will become apparent later in this work by time resolving the optical field emission.

## Homochromatic attosecond streaking

As vacuum is dispersive to electron pulses, a temporal characterization has a concrete meaning at a specific point in space. Because the generated electron pulses scrutinize the ‘sample’ during the recollision with the parent surface as to their temporal structure at this point, it is mostly relevant for harnessing the power of these pulses in time-resolved applications.

**Fig. 2:**
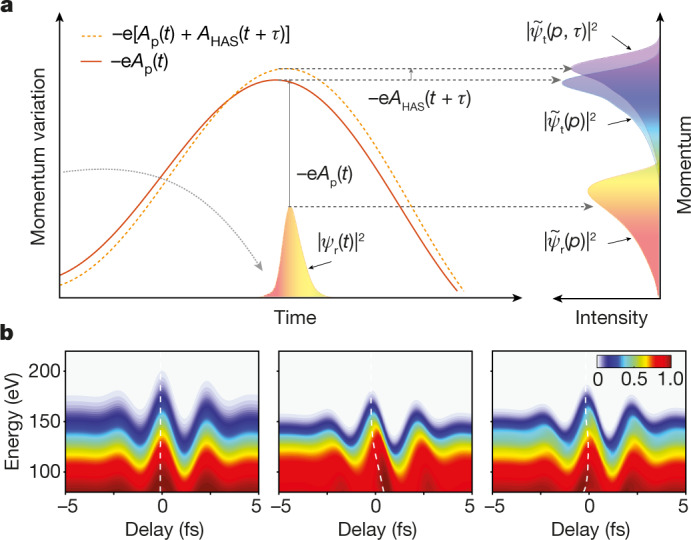
Homochromatic attosecond streaking. **a**, An electron wave packet *ψ*_r_(*t*), sampled on recollision at the nanotip surface, is represented by its momentum distribution ${\widetilde{\psi }}_{{\rm{r}}}(p)$and corresponding spectral intensity ${\left|{\widetilde{\psi }}_{{\rm{r}}}(p)\right|}^{2}$. Following backscattering, further acceleration of the wave packet by the vector potential of the pump field *A*_p_(*t*) (red curve) gives rise to a terminal wave packet ${\widetilde{\psi }}_{{\rm{t}}}(p)$ with corresponding spectral intensity $I(p)={\left|{\widetilde{\psi }}_{{\rm{t}}}(p)\right|}^{2}$. The coherent superposition (dashed yellow curve) of the pump field with a weak, time-delayed (*τ*) replica (the gate) results in a momentum shift, −e*A*_HAS_(*t* + *τ*) of the final momentum distribution $I(p,\tau )={\left|{\widetilde{\psi }}_{{\rm{t}}}(p,\tau )\right|}^{2}$. **b**, Simulated delay-dependent homochromatic attosecond streaking (HAS) spectrograms for electron pulses without chirp (left panel), positive chirp of 3.5 × 10^3^ as^2^ (middle panel) and third-order chirp of 1 × 10^5^ as^3^ (right panel). The dashed white curves highlight the resulting energy-dependent shifts of the intensity modulation. Source data

To understand how, we revisit the process of strong field recollision of an electron wave packet under an intense optical waveform (Fig. 2). Set free around the peak of a laser field crest, an electron pulse will recollide with the tip surface at an instance *t*_r_ (Fig. 2a) with an energy of about 3*U*_p_ (refs. ^11,37^). An attosecond measurement of the electron pulse entails access into its waveform *ψ*_r_(*t*) at the surface of the nanotip or equivalently, into its associated complex spectral amplitude ${\widetilde{\psi }}_{{\rm{r}}}(p)$, in which *p* is the recollision momentum of the electron. However, as ${\widetilde{\psi }}_{{\rm{r}}}(p)$ is not directly accessible in measurements, it is important to link it to other measurable quantities. Following backscattering off the tip surface, the wave packet acquires further phase from both its interaction with the driving field (Volkov phase) as well owing to its free-space propagation. If we define an auxiliary terminal wave packet ${\widetilde{\psi }}_{{\rm{t}}}(p)$, the spectral intensity $I(p)={| {\widetilde{\psi }}_{{\rm{t}}}(p)| }^{2}$ (refs. ^38–40^) (as marked in Fig. 2a) can be expressed as (in atomic units):

1 $$I(p)={|{\widetilde{\psi }}_{t}(p)|}^{2}={\left|{\int }_{-{\rm{\infty }}}^{{\rm{\infty }}}{\psi }_{r}({t}_{r}){\rm{\exp }}\left[i\frac{{p}^{2}}{2}{t}_{r}\right]{\rm{\exp }}[-{iS}(p,{t}_{r}{\rm{;}}{A}_{p}(t))]{\rm{d}}{t}_{r}\right|}^{2}$$

in which $S(p,{t}_{{\rm{r}}}\,;{A}_{{\rm{p}}}(t))={\int }_{{t}_{{\rm{r}}}}^{{\rm{\infty }}}\left[\frac{1}{2}\right.{[p+{A}_{{\rm{p}}}(t)]}^{2}-\left.\frac{1}{2}{p}^{2}\right]\,{\rm{d}}t$ denotes the Volkov phase imparted to the electron wave packet by the vector potential *A*_p_(*t*) of the intense driving pulse (hereafter referred to as pump, see red curve in Fig. 2a) after rescattering at time *t*_r_. Hence reconstruction of the recolliding wave packet *ψ*_r_(*t*) should be possible if, other than *I*(*p*), which is a directly measurable quantity (that is, the spectrum of the electron emission), the phase of ${\widetilde{\psi }}_{{\rm{t}}}(p)$ as well as the waveform *A*_p_(*t*) are accessed.

Access to the phase of ${\widetilde{\psi }}_{{\rm{t}}}(p)$ is possible by temporally gating the electron emission with a weak replica of the pump pulse (hereafter referred to as gate with vector potential *A*_g_(*t*)) when $\eta \equiv {\left|{A}_{{\rm{g}}}(t)\right|}^{2}/{\left|{A}_{{\rm{p}}}(t)\right|}^{2}\ll 1$ (refs. ^27,31^). In this limit, the pump pulse is solely responsible for releasing the electron wave packet, whereas the gate pulse primarily alters its phase. This is manifested by the shift and reshaping of the terminal photoelectron spectra at the end of the driving pulse (Fig. 2a). If the delay *τ* between pump and gate pulses is varied (see Methods), the terminal spectral distribution of the released electron, $I(p,\tau )={\left|{\widetilde{\psi }}_{{\rm{t}}}(p,\tau )\right|}^{2}$ can be approximated as:

2 $$I(p,\tau )\propto {\left|{\int }_{-{\rm{\infty }}}^{{\rm{\infty }}}{\psi }_{t}\left({t}_{r}\right){\rm{\exp }}\left[i\frac{{p}^{2}}{2}{t}_{r}\right]{\rm{\exp }}\left[-{iS}\left(p,{t}_{r}{\rm{;}}{A}_{{HAS}}\left(t\right)\right)\right]{\rm{d}}{t}_{r}\right|}^{2}$$

in which *ψ*_t_(*t*) is the inverse Fourier transform of ${\widetilde{\psi }}_{{\rm{t}}}(p)$ and *A*_HAS_(*t* + *τ*) represents an effective vector potential, which is explicitly related to the incident vector potential *A*_g_(*t*) of the gate pulse as shown in Methods and which accounts for the momentum an electron accumulates from the instance of its birth to the detection. Equation (2) implies: (i) a variation of *τ* permits the composition of a streaking-like spectrogram whose reconstruction can allow retrieving the phase of ${\widetilde{\psi }}_{{\rm{t}}}(p)$ and (ii) the momentum variation of the electron distribution follows *A*_HAS_(*t*). Whereas implication of (ii) (see Methods) allows sampling of the waveform of a light pulse^31^, (i) is essential for mapping the dynamics of the field emission. To distinguish from conventional attosecond streaking, we refer to this approach as homochromatic attosecond streaking (HAS), highlighting that the carrier frequency of the pump and gate fields is identical.

Shown in Fig. 2b are simulated HAS spectrograms under conditions pertinent to the experiments presented here. Notably, and in close analogy to ordinary attosecond streaking^41,42^, different types of chirp of the recolliding electron pulse *ψ*_r_(*t*) yield distinct visual signatures in the spectrogram, manifested as shifts of the spectral intensity modulation versus delay and energy, as highlighted by the dashed white curves in Fig. 2b.

**Fig. 3:**
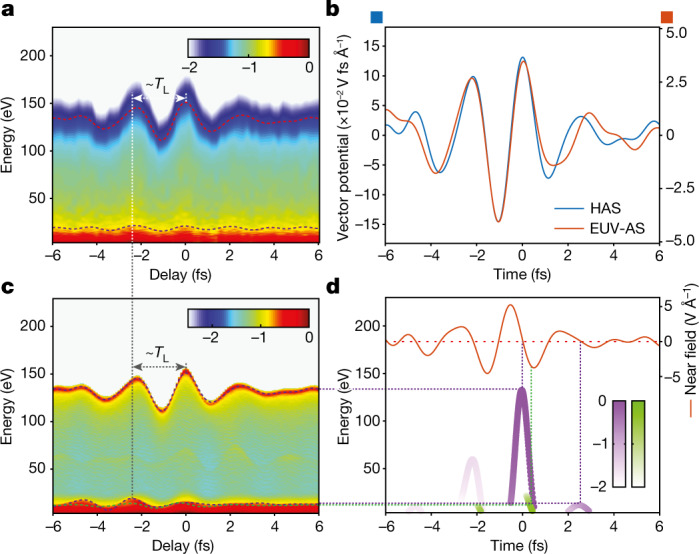
Homochromatic attosecond streaking of the optical field emission. **a**, Homochromatic attosecond streaking (HAS) spectrogram of the optical field emission from a tungsten nanotip consisting of 60 individual electron spectra recorded as a function of the delay (step size of 200 as) between an intense (*I*_p_ ≈ 25.4 TW cm^−2^) single-cycle pulse and a 6.3 × 10^−3^ times weaker gate pulse. **b**, Vector potential *A*_g_(*t*) evaluated by the photoelectron energy cutoff variation in the HAS spectrogram in panel **a** as a function of the delay (blue) and from an ordinary streaking spectrogram (red) (see Extended Data Fig. 9) in absolute units. **c**, Simulated HAS spectrogram using the experimentally recorded field waveforms of pump and gate pulses. The dashed vertical lines in **a** and **c** denote different rescattering events. **d**, Terminal energies of direct (green) and backscattered electron emissions (purple) associated (dashed horizontal lines) with corresponding cutoff energies in the spectrogram (**c**), as well as release times (dashed vertical lines) near-field pump waveform (red curve). EUV-AS, extreme ultraviolet attosecond streaking. Source data

In our experiments, we derived pump and gate pulses by the spatiotemporal division of the sub-cycle optical transients using a dual-mirror module as shown in Fig. 1a. Figure 3a plots a HAS spectrogram recorded by our apparatus. A ratio *η* ≈ 6.3 × 10^−3^ warrants that the gate pulse is sufficiently weak to serve as a phase gate (see Methods), whereas a remaining weak amplitude modulation (5–10%) of the spectrogram versus delay was useful for evaluating the timing between pump and gate pulses, and therewith to clock the recollision instance with respect to the waveform of the driving pump pulse.

The evaluated *A*_g_(*t*) (blue curve in Fig. 3b) by HAS exhibits an excellent waveform matching that derived by EUV attosecond streaking (red curve in Fig. 3b), as attested by the degree of similarity^43^ (around 0.95). In this case, the HAS-evaluated vector potential represents the near field of the gate pulse in the vicinity of the tip. Moreover, the ratio between the absolute amplitudes of the vector potentials evaluated by the two methods provides a direct measurement of the field-enhancement factor *f* = 3.73 ± 0.25, which is in close agreement with the result of the methodology highlighted in Fig. 1c (conducted with a different nanotip) and the theoretical estimations.

How time-domain measurement of electron emission in nanostructured materials benefits the intuitive understanding of the process and its real-time clocking can be best appreciated by a close inspection of the oscillating phases of high and low cutoff energies in Fig. 3a. For instance, an apparent delay of the maxima of the corresponding oscillations (highlighted by red and blue dashed curves, respectively, in Fig. 3a) versus delay indicates a retardation in the electron emission at lower energies by approximately a laser cycle (*T*_L_ ≈ 2.3 fs). This feature, also well reproduced in our simulation (Fig. 3c), shows that the low-energy emission consists of a mixture of direct electrons emerging within the main half-cycle of the driving field (green dots in Fig. 3d) and low-energy backscattered electrons generated approximately a cycle of the driving field later (purple dots in Fig. 3d). Moreover, the uniform amplitude and energy modulation of the photoelectron spectrum (Fig. 3a) over a broad range of energies (50–150 eV) offers compelling evidence that emission is confined to a single electron pulse generated within a field half-cycle.

**Fig. 4:**
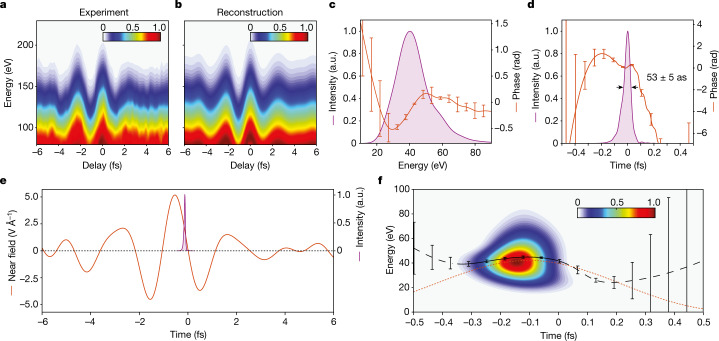
Measurement of attosecond electron pulses in the optical field emission. **a**,**b**, Experimentally recorded (**a**) and reconstructed (**b**) HAS spectrograms from a W nanotip. **c**, Retrieved backscattered electron-pulse spectrum (magenta fill) and its spectral phase (red curve). **d**, Intensity profile of the electron pulse (magenta fill) and its temporal phase (red line). **e**, Near field (red curve) and its timing with respect to the attosecond electron pulse (magenta fill). **f**, Time–frequency analysis of the attosecond electron pulse (false colour is intensity in arbitrary units) and retrieved release times (black curve). The dashed red curve denotes release times calculated semiclassically for the near-field light waveform in **e**. Error bars represent standard deviations of the corresponding values indicated. Source data

For the reconstruction of the electron wave packet, we are primarily interested in the properties of the recolliding electron pulses at energies typically higher than 20 eV, that is, where this pulse could serve high-resolution/atomic-scale, spatial analysis of surfaces^28^. Considering the ratio of the terminal energy of electrons (about 10*U*_p_) and that at the recollision instance (about 3.17*U*_p_), we conclude that the relevant information is encoded at the high-energy end (>80 eV) of the spectrogram of Fig. 3a, as isolated in Fig. 4a. Figure 4b shows the numerical reconstruction of data in Fig. 4a based on equations (1) and (2), the retrieved field parameters *A*_p_(*t*) and *A*_g_(*t*) (Fig. 3b), the absolute time delay *τ* (Extended Data Fig. 8) and the numerical algorithm detailed in Methods.

The retrieved electron-pulse profiles in spectral and time domains are shown in Fig. 4c,d, respectively. The spectrum extends over the range 20–80 eV (Fig. 4c, magenta-shaded curve) and is temporally confined to about 53 ± 5 as, as measured at the full width at half maximum of its intensity profile (Fig. 4d, magenta-shaded curve). Figure 4e compares the retrieved electron pulse and the near-field profiles, and shows that the electron wave packet revisits the surface of the nanotip (magenta-shaded curve) at times close to the zero transition of the driving field (red curve). This observation is compatible with the semiclassical understanding of strong-field rescattering in atoms^11,36^.

A closer inspection of the retrieved spectral and temporal phases (red curves in Fig. 4c,d) also shows a negligible temporal spread of the electron pulse compared with its Fourier-limited duration (about 50 as). The compatibility of this finding with the semiclassical model can be best appreciated by comparing the group delay of the electron release (solid black line in Fig. 4f) as evaluated by the time–frequency analysis (Fig. 4f, false colour) of the attosecond electron-pulse waveform in Fig. 4d with the semiclassical release times (dashed red curve in Fig. 4f) calculated using the near-field waveform of the driving pulse (red curve in Fig. 4e). The comparison highlights that, as anticipated by the semiclassical recollision model, electrons of energy close to the cutoff, that is, where short and long trajectories merge, carry a minor temporal chirp. This feature, common in near-cutoff high harmonic emission in atoms^24,41^, further strengthens the link between strong-field optics in atoms and nano-optics systems.

## Conclusion

Direct measurement of attosecond electron pulses in the optical field emission extends the repertoire of ultrafast-science tools. The technique presented here could offer a broad range of capabilities for tracking ultrafast collective or correlated electron dynamics in various materials deposited or attached to tungsten nanotips^44,45^, as well as in gas-phase systems. The recolliding electron pulses shall also enable attosecond-resolved, laser-induced electron diffraction^46^ experiments at nanotips, as well as nanodiffraction ex situ experiments in specimens placed at nanometric distance from the tip apex^47^. Such possibilities could enable new routes for exploring the structure and dynamics of condensed matter in four dimensions.

## Methods

### Experimental

#### Attosecond EUV streaking

For attosecond EUV streaking measurements (Extended Data Fig. 1a), the sub-cycle transients are focused onto the neon gas jet to generate EUV pulses by high-harmonic generation. The collinearly propagating EUV and optical pulses are spatially separated by a Zr filter, which also acts as an EUV high-pass spectral filter (>70 eV), enabling the isolation of a single attosecond pulse. The EUV and optical pulses are reflected off a dual-mirror assembly, which consists of a Mo/Si inner mirror (centred at around 85 eV) and a nickel outer mirror, respectively. Inner and outer mirrors can be delayed with nanometric resolution (Extended Data Fig. 1a). EUV and optical pulses are focused onto a second Ne gas jet. Photoelectron spectra recorded as a function of the delay between the inner and outer mirrors allow the composition of attosecond streaking spectrograms, which allow the detailed characterization of the attosecond EUV pulse and, notably for these experiments, the field waveform of the optical pulse. Details about the relevant techniques can be found in refs. ^35,41,48^.

#### HAS

**Extended Data Fig. 3:**
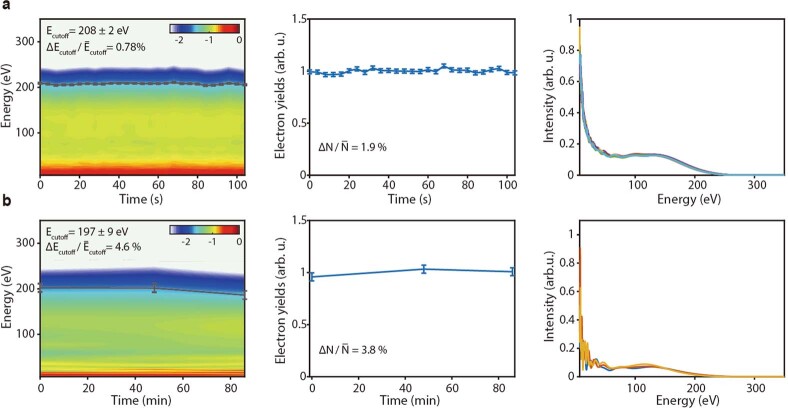
Stability of electron emission from tungsten nanotips. **a**, Consecutive measurements of photoelectron spectra recorded under identical conditions and over a time range of about 100 s (left). The grey line indicates the cutoff energy. Electron yield evaluated by integration of the individual spectra in the left panel (middle). Individual spectra superimposed for comparison (right). **b**, The same measurement as in **a** but over a time period of about 80 min. Source data

HAS measurements are performed on the same setup by means of (1) automated removal of the Ne gas used to generate high harmonics and the Zr filter, (2) automated replacement of the inner mirror in the dual-mirror module by a Ni-coated one of the same focal lengths (Extended Data Fig. 1b) and (3) the streaking gas nozzle is replaced by an electrically grounded tungsten nanotip. The above setup modifications are executed in a fraction of a minute and warrant identical conditions for all relevant measurements. In the HAS configuration of the setup in Extended Data Fig. 1b, Ni-coated inner and outer mirrors spatially divide the optical pulse into pump (inner mirror beam) and gate (outer mirror) pulses. A delay between the pulses reflected off the inner and outer mirrors, respectively, is introduced by a precision transitional stage (see inset in Extended Data Fig. 1b). The HAS measurements as well as the intensity-scaling measurements of the electron spectrum and yields were performed with driving-field intensities below a critical intensity, at which the irreversible optical damage of the nanotips occurs. This upper limit of intensity was experimentally identified by observing sudden and irreversible drops of electron cutoff energy in intensity-scaling measurements.

#### Measurement of the absolute electron yield in the optical field emission

For the measurement of the total electron counts per pulse generated in our setup, a thin electrode (size roughly 5 mm × 5 mm) is introduced about 2 mm above the nanotip (Extended Data Fig. 2). This configuration allows detection of released electrons over a solid angle *Ω* > π steradians. The induced voltage on the thin plate is measured by a lock-in amplifier at the reference frequency of the repetition rate of the driving laser (about 3 KHz). The electronic current is evaluated by dividing the induced voltage by the system impedance (10 MΩ). The total electron count per pulse is in turn obtained by dividing the current by the repetition rate of the laser and the electron charge.

#### Sample robustness and measurement stability study

Tungsten nanotips were exposed to intensities of up to *I* ≈ 45 TW cm^−2^ without noticing any damage. This was verified by performing the intensity–cutoff energy study shown in Fig. 1b, of both increasing as well as decreasing intensity, and recorded identical curves. When the critical intensity reached *I* ≈ 45 TW cm^−2^, the tip is damaged and the cutoff energy irreversibly drops to much lower values without the possibility for recovery unless a new tip is installed.

To explore short-term and long-term stability of our system, we recorded electron spectra as a function of time under identical conditions and for intensities typically higher than those used in the HAS measurements. Data shown in Extended Data Fig. 3 suggest an excellent stability of cutoff energy and electron yield, implying the structural robustness of the nanotip on the timescale of typical measurements (a few minutes) as well as over several hours.

### One-dimensional, semiclassical simulations of the optical field emission

The time-dependent ionization probability from a tungsten nanotip was approximated by the Fowler–Nordheim formula as^5,17,49,50^:

3 $$p(E(t))=N\theta (-E(t)){\left|E(t)\right|}^{2}\,\exp \,\left[-\frac{4\sqrt{2m}{\phi }^{3/2}}{3\hbar e\left|E(t)\right|}\right],$$

in which *E*(*t*) is the electric-field waveform of the driving pulses, *ϕ* is the work function of the metal and *m*, *ħ* and *e* are electron mass, reduced Planck’s constant and electron charge, respectively. We calculated electron trajectories using the classical equations of motion in the single-electron limit as^7,8,11,17,36^:

4 $$m\frac{{{\rm{d}}}^{2}{z}_{i}}{{\rm{d}}{t}^{2}}=-e{f}_{0}E(t)$$

Here *i* is the index of each individual trajectory and *f*_0_ is the field-enhancement factor. At the end of the pulse, an electron spectrum is evaluated by a spectral binning of the energies of all trajectories weighted by the ionization rate at the instances of their births.

**Extended Data Fig. 4:**
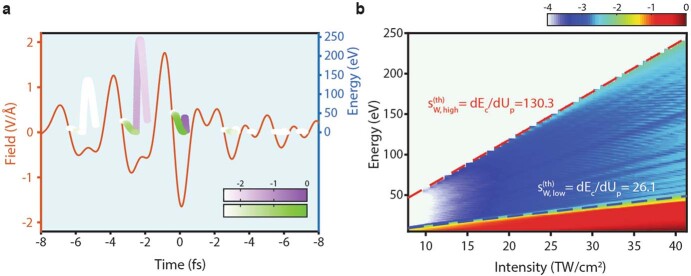
Semiclassical simulations of the optical field emission from tungsten nanotips in the single-cycle limit. **a**, Terminal electron energy as a function of the ionization time under the electric field of the incident single-cycle pulses (red curve). A peak field intensity of 41 TW cm^−2^ was used in accordance with the experiments. Purple and green lines indicate terminal energies of backscattered and direct electrons as functions of birth time, for which the ionization rate is encoded on the line shading in logarithmic scale. **b**, Electron spectra plotted as a function of the peak field intensity. Red and blue dashed lines indicate the high and low cutoff energies, respectively. Source data

For the experiments described here, we simulated electron spectra from tungsten (*ϕ* = 4.53 eV) versus peak field intensity of the driving pulse (Extended Data Fig. 4). The driving field (red curve in Extended Data Fig. 4a) used in our simulations was measured by an EUV attosecond streaking setup^35,48^ integrated in our apparatus. A field-enhancement factor of *f*_0_ = 3.46 used in these simulations was derived experimentally as described in the main text. The quiver length of electrons in our intensity range (the longest length of about 1.7 nm at the highest intensity of about 41 TW cm^−2^) is considerably shorter than the decay length of the near field (about 30 nm). Hence the released electrons experience a nearly homogeneous near field and therefore quenching effects^17^ owing to the near-field decay can be neglected.

In agreement with the data of Fig. 1c, the simulated electron spectra exhibit two well-discernible energy cutoffs (red and blue dashed lines in Extended Data Fig. 4b) associated with the backscattered (purple line in Extended Data Fig. 4a) and the direct (green line in Extended Data Fig. 4a) electrons. The slopes of high (${s}_{{\rm{W}},{\rm{high}}}^{({\rm{th}})}={{\rm{d}}E}_{{\rm{c}}}/{{\rm{d}}U}_{{\rm{p}}}=130.3$) and low (${s}_{{\rm{W}},{\rm{low}}}^{({\rm{th}})}=26.1$) cutoff energies agree well with those in our measurements (Fig. 1c). The theory shows further emission cutoffs at energies lower than that of the direct electrons. Because these are relatively weak, they do not leave any direct signatures in the photoelectron spectra. Yet such contributions become visible in HAS spectrograms, as discussed in Fig. 3.

### FDTD simulations of the field enhancement

**Extended Data Fig. 5:**
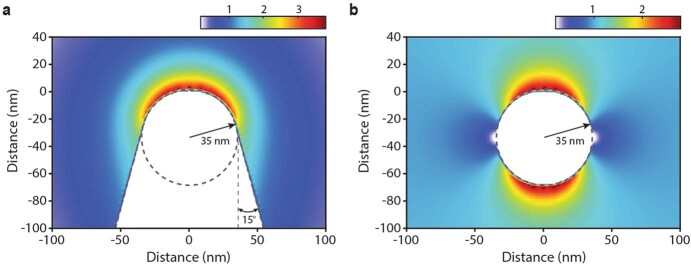
FDTD simulations of the near-field enhancement. Evaluated spatial distribution of the field-enhancement factor at the apex of a tungsten nanotip with an apex radius of 35 nm and opening angle of 15° (**a**) and a similarly sized tungsten sphere (**b**). Source data

To theoretically estimate the near-field enhancement in the vicinity of the tungsten nanotip, we numerically solved Maxwell’s equations by means of three-dimensional finite-difference time-domain (FDTD) simulations. The nanotip was modelled as shown in Extended Data Fig. 5a as a sphere with radius of 35 nm that smoothly transitions to a cone with an opening angle (single side) of 15° and considering optical properties for tungsten^51^. The simulations predict a peak field-enhancement factor of about 3.8 close to the surface at the tip apex. For comparison, the spatial distribution of the enhancement at a respective tungsten nanosphere (that is, excluding the cone) is shown in Extended Data Fig. 5b, with a slightly lower peak enhancement factor of approximately 2.7.

### Three-dimensional, semiclassical trajectory simulation including charge interaction

**Extended Data Fig. 6:**
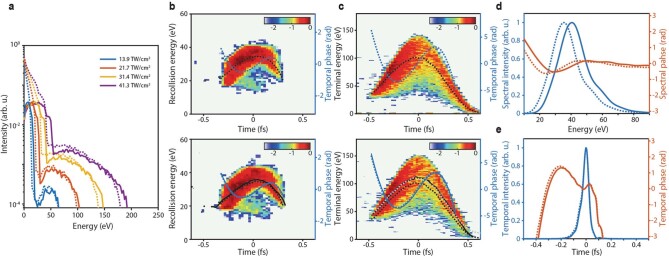
Impact of charge interactions on electron emission dynamics. **a**, Electron spectra obtained from three-dimensional semiclassical M^3^C trajectory simulations for the experimental parameters without (dashed curves) and with (solid curves) the inclusion of charge interactions for representative pulse peak intensities. **b**, Recollision energy distribution as a function of the release time without (top) and with (bottom) charge interactions (false colour denotes spectral intensity). Black and blue curves are the instantaneous energy and the temporal phase, respectively, whereas dashed and solid curves mark the cases without and with the charge interactions, respectively. **c**, The same analysis as in **b** but for the terminal energy of the electron ensemble. The charge interaction free instantaneous energy (dashed black curve) is virtually identical to that modified by charge interactions (solid black curve), except for a uniform shift (dashed grey curve) along the energy axis. **d**, Retrieved spectral intensity (blue) and phase (red) of the recolliding electron pulse with (dashed curves) and without (solid curves) inclusion of space–charge effects on the terminal electron pulse. **e**, The same analysis as in **d** but in the temporal domain. Source data

To inspect whether charge interaction substantially affects the electron emission dynamics for the considered parameters, we performed semiclassical trajectory simulations using the Mie–mean field–Monte Carlo (M^3^C) model^52^. The latter has been used extensively for the study of strong-field ionization in dielectric nanospheres^53–55^ and has recently also been adopted for the description of metallic nanotips^56^. In brief, we mimic the apex of the nanotip as one-half of a sphere with corresponding radius. The near field is evaluated as the combined linear polarization field owing to the incident pulse (evaluated by means of the Mie solution of Maxwell’s equations) and an extra nonlinear contribution owing to charge interaction treated as a mean field in electrostatic approximation (evaluated by high-order multipole expansion). The latter includes Coulomb interactions among the emitted electrons as well as their image charges (that is, an extra sphere polarization caused by the free electrons). Photoelectron trajectories are generated by Monte Carlo sampling of ionization events at the sphere surface, at which we evaluate tunnelling probabilities within WKB approximation by integration through the barrier provided by the local near field. Trajectories are propagated in the near field by integration of classical equations of motion and accounting for electron–atom collisions through respective scattering cross-sections for electrons moving within the material. To mimic the slightly higher peak enhancement of the linear response near field at a tungsten tip (≈3.8) as compared with a sphere (≈2.7), see Extended Data Fig. 5, we rescaled the incident laser intensity by a factor of 1.4. The performed M^3^C simulations predict about three times fewer emitted electrons than the experiments, which we attribute to contributions of slow electrons originating from the shank of the nanotip. This is substantiated by comparison of the total electron yields predicted for the nanotip and the half-sphere, obtained through integration of the local ionization rates over the respective surface areas and the pulse duration. However the charge densities at the pole of the sphere and the tip apex are comparable, enabling to inspect the impact of charge interactions within the simplified simulation model. Extended Data Fig. 6a compares multielectron spectra simulated with (solid curves) and without (dashed curves) accounting for charge interactions among the individual electrons and for four representative settings of the peak intensities (and corresponding electron yields) of the driving field, as indicated in the legend. The presence of charge interaction is primarily manifested by a noticeable decline of the yield of direct electrons (<50 eV), which—in accordance with earlier works^52,57^—can be attributed to a partial trapping of these electrons in the vicinity of the tip. Quasi-static electric fields generated by the trapping of low-energy electrons in turn affect the dynamics of the recolliding electrons and give rise to an increase of the terminal electron-energy cutoff^52,57^. For the highest intensity and corresponding electron yield, this shift is approximately 8% in energy.

Although transparent manifestations of such effects cannot be discerned in our experiments, it is useful to understand potential implications of charge interactions on the characterization of attosecond electron pulses using HAS. To this end, we extended our analysis to the time domain for pulses of intensity (about 31 TW cm^−2^) and electron yield (about 600 electrons per pulse), mimicking the experimental conditions in our HAS measurements.

Extended Data Fig. 6b shows the recollision energy distribution of the electron ensemble versus release times without (top) and with (bottom) inclusion of charge interactions. The instantaneous release energy is evaluated by taking the first momentum of the time-resolved spectra (black dashed and solid curves) and the temporal phases (blue dashed and solid curves) by the temporal integration of the instantaneous energy. Their comparison, shown in Extended Data Fig. 6b, suggests marginal differences and, thus, corresponding negligible effects on the temporal characteristics of the electron pulse at the time of recollision.

When the time-domain analysis is extended to the terminal energies of the recolliding electrons versus release time, the space–charge interactions are manifested by a uniform upshift of terminal energies by about 8% but leave the temporal phase of the terminal electron wave packet unaffected (Extended Data Fig. 6c).

To investigate how this energy shift could potentially affect the retrieval of the electron pulse at the instance of recollision, we applied the simulated phase effects on our experimental data (see the section ‘HAS reconstruction methodology’) and evaluated once again the spectral and temporal properties of the recolliding attosecond electron pulse. Key observations associated with space–charge interactions include a weak, uniform shift of the central energy of the recolliding electron by a few electronvolts (Extended Data Fig. 6d) and a subtle change in the temporal profile of the electron pulse (Extended Data Fig. 6e), resulting in an approximately 4-as elongation of its duration at the full width at half maximum, which is within the error of the experimental reconstruction (about 5 as).

### Mathematical formulation of HAS

The key objective of HAS is to retrieve the temporal structure of an attosecond electron pulse wave packet *ψ*_r_(*t*) at the moment of its recollision on its parent surface. As this wave function is not directly accessible, it is necessary to link it to other quantities that are either directly measured in the experiments (such as the terminal spectral intensity $I(p)={\left|{\widetilde{\psi }}_{{\rm{t}}}(p)\right|}^{2}$ at a detector) or can be reconstructed from the experimental data.

#### Description of strong-field electron emission

Considering an electron released from and driven back to a surface by a strong pump field *E*_p_(*t*), its recolliding wave packet *ψ*_r_(*t*) can be linked to its terminal spectral amplitude ${\widetilde{\psi }}_{{\rm{t}}}(p)$ at the end of interaction with the driving pulse. The time-dependent recollision wave packet *ψ*_r_(*t*) is expressed by means of its Fourier representation ${\widetilde{\psi }}_{{\rm{r}}}(p)={\int }_{-\infty }^{\infty }{\psi }_{{\rm{r}}}({t}_{{\rm{r}}})\exp [{\rm{i}}\frac{{p}^{2}}{2}{t}_{{\rm{r}}}]{\rm{d}}{t}_{{\rm{r}}}$ and, following the recollision, the spectral amplitude is transformed to the terminal form^38–40,58^ (in atomic units):

5 $${\widetilde{\psi }}_{{\rm{t}}}(p)\propto {\rm{i}}{\int }_{-\infty }^{\infty \,}{\psi }_{{\rm{r}}}({t}_{{\rm{r}}})\exp \,\left[{\rm{i}}\frac{{p}^{2}}{2}{t}_{{\rm{r}}}\right]\,\exp [-{\rm{i}}S(p,\infty ,{t}_{{\rm{r}}}{\rm{;}}{A}_{{\rm{p}}}(t))]{\rm{d}}{t}_{{\rm{r}}}$$

Here *S* is the Volkov phase imparted to the electron wave packet only by the vector potential *A*_p_(*t*) of the pump pulse after recollision at an instance *t*_r_, at which the general form of the Volkov phase accumulated from a time instance *t*_1_ to a later instance *t*_2_ by an electric field with vector potential *A*(*t*) is expressed as^59^:

6 $$S(p,{t}_{2},\,{t}_{1}{\rm{;}}A(t))={\int }_{{t}_{1}}^{{t}_{2}}\left[\frac{1}{2}\right.{\left[p+A(t)\right]}^{2}-\left.\frac{1}{2}{p}^{2}\right]{\rm{d}}t$$

Note that equation (6) excludes free-space propagation, that is, it vanishes in the absence of the field, and equation (5) reflects the momentum-dependent wave function at the surface, including phases accumulated only by the pump field.

Earlier semiclassical theories of strong-field emission^39,40,58^ in atoms have suggested that the recolliding wave packet *ψ*_r_(*t*) can be expressed by integration over ionization instance *t*′ before recollision at time *t*_r_ and over canonical momenta *p*′ in terms of the ionization amplitude, dictated by the dipole transition *E*_p_(*t*′)*d*(*p*′ *+* *A*_p_(*t*)), the scattering amplitude typically described as *g*(*p*′ *+* *A*_p_(*t*_r_)) and the Volkov phase that the electron accumulates from *t*′ to *t*_r_ as:

7 $${\psi }_{{\rm{r}}}({t}_{{\rm{r}}})={\int }_{-\infty }^{{t}_{{\rm{r}}}}\int g({p}^{{\prime} }+{A}_{{\rm{p}}}({t}_{{\rm{r}}})){E}_{{\rm{p}}}({t}^{{\prime} })d({p}^{{\prime} }+{A}_{{\rm{p}}}({t}^{{\prime} }))\times \exp \,[-{\rm{i}}S({p}^{{\prime} },{t}_{{\rm{r}}},{t}^{{\prime} }{\rm{;}}{A}_{{\rm{p}}}(t))-{\rm{i}}\frac{{p}^{2}}{2}({t}_{{\rm{r}}}-{t}^{{\prime} })+{\rm{i}}\phi {t}^{{\prime} }]{\rm{d}}{p}^{{\prime} }{\rm{d}}{t}^{{\prime} }$$

Here *d* and *g* are the dipole and scattering matrix element, respectively, as defined in refs. ^39,40,58^, and e^i*ϕt*^ reflects the extra phases acquired during the time evolution of the bound state before ionization. In our experimental setting of HAS in which the Keldysh parameter of *γ* ≈ 0.38 suggests the tunnelling regime^60^, those three processes (ionization, propagation and backscattering) can be independently treated without non-adiabatic corrections on the Volkov phase, *S* (refs. ^61,62^).

#### Description of electron wave packets under addition of a weak gate field

Equation (5) implies that access to *ψ*_r_(*t*) is possible if ${\widetilde{\psi }}_{{\rm{t}}}(p)$ and *A*_p_(*t*) are known. Therefore our goal is to describe how these quantities can be accessed using a phase-gating process of the optical field emission by a weak replica of the driving pulse (gate pulse). Now we inspect the effects of adding the gate pulse on the dynamics of the electron described in equations (5)–(7). We define the gate pulse by a field *E*_g_(*t* + *τ*) and its vector potential *A*_g_(*t* + *τ*), in which *τ* is the delay between the pump and gate pulses as described above. By replacing the pump fields and its vector potentials by the superposition of pump and gate pulses in equations (5)–(7) as *E*_p_(*t*) → *E*_p_(*t*) + *E*_g_(*t* + *τ*) and *A*_p_(*t*) → *A*_p_(*t*) + *A*_g_(*t* + *τ*), the terminal spectral amplitude perturbed by the gate can be rewritten as:

8 $${\widetilde{\psi }}_{{\rm{t}}}(p,\tau )\propto {\rm{i}}{\int }_{-\infty }^{\infty }{\psi }_{{\rm{r}}}^{({\rm{g}})}({t}_{{\rm{r}}},\tau )\exp \left[{\rm{i}}\frac{{p}^{2}}{2}{t}_{{\rm{r}}}\right]\,\exp \left[-{\rm{i}}S\left(p,\infty ,{t}_{{\rm{r}}};{A}_{{\rm{p}}}(t)+{A}_{{\rm{g}}}(t+\tau )\right.\right]{\rm{d}}{t}_{{\rm{r}}}$$

in which ${\psi }_{{\rm{r}}}^{({\rm{g}})}({t}_{{\rm{r}}},\tau )$ denotes the recolliding electron wave packet perturbed by the extra gate pulse as marked by the superscript (g) to be distinguished from the gate-free counterpart *ψ*_r_(*t*) (see equation (7)). Because the gate-free quantity *ψ*_r_(*t*) is of interest in this discussion, the subject in this section is how to express ${\widetilde{\psi }}_{{\rm{t}}}(p,\tau )$ in terms of *ψ*_r_(*t*) with phase terms introduced by the gate.

First we investigate the perturbed recolliding electron wave packet $${\psi }_{{\rm{r}}}^{({\rm{g}})}({t}_{{\rm{r}}},\tau )$$ in equation (8) and how to link it with the gate-free electron wave packet *ψ*_r_(*t*). If the gate field is sufficiently weak, that is, $$\eta ={\left|{A}_{{\rm{g}}}(t)\right|}^{2}/{\left|{A}_{{\rm{p}}}(t)\right|}^{2}\ll 1$$, the dipole transition and scattering amplitudes can be considered invariant, that is, $$\left[{E}_{{\rm{p}}}(t)+{E}_{{\rm{g}}}(t+\tau )\right]d(p+{A}_{{\rm{p}}}(t)\,+$$$${A}_{{\rm{g}}}(t+\tau ))\approx {E}_{{\rm{p}}}(t)g(p+{A}_{{\rm{p}}}(t))$$ and $$g(p+{A}_{{\rm{p}}}(t)+{A}_{{\rm{g}}}(t+\tau ))\approx g(p+{A}_{{\rm{p}}}(t))$$ in the expression of the recolliding electron wave packet (equation (7)). In such case, the gate only modifies the phase imparted on the wave packet between ionization and recollision:

9 $${\psi }_{{\rm{r}}}^{({\rm{g}})}({t}_{{\rm{r}}},\tau )\approx {\int }_{-\infty }^{{t}_{{\rm{r}}}}{\rm{d}}{t}^{{\prime} }\int {\rm{d}}{p}^{{\prime} }g({p}^{{\prime} }+{A}_{{\rm{p}}}({t}_{{\rm{r}}})){E}_{{\rm{p}}}({t}^{{\prime} })d({p}^{{\prime} }+{A}_{{\rm{p}}}({t}^{{\prime} }))\times \exp \,\left[-{\rm{i}}S({p}^{{\prime} },{t}_{{\rm{r}}},{t}^{{\prime} }{\rm{;}}{A}_{{\rm{p}}}(t)+{A}_{{\rm{g}}}(t+\tau ))-{\rm{i}}\frac{{p}^{2}}{2}({t}_{{\rm{r}}}-{t}^{{\prime} })+{\rm{i}}\phi {t}^{{\prime} }\right]$$

In view of these considerations, the variation of the phase (see equation (6)) can be expressed as:

10 $$\begin{array}{l}S(p,{t}_{2},{t}_{1};{A}_{p}(t)+{A}_{g}(t+\tau ))={\int }_{{t}_{1}}^{{t}_{2}}[\frac{1}{2}{[p+{A}_{p}(t)+{A}_{g}(t+\tau )]}^{2}-\frac{1}{2}{p}^{2}]{\rm{d}}t\\ \approx \mathop{\underbrace{{\int }_{{t}_{1}}^{{t}_{2}}[\frac{1}{2}{[p+{A}_{p}(t)]}^{2}-\frac{1}{2}{p}^{2}]{\rm{d}}t}}\limits_{=S(p,\,{t}_{2},{t}_{1};{A}_{p}(t))}+\mathop{\underbrace{{\int }_{{t}_{1}}^{{t}_{2}}[p+{A}_{p}(t)]{A}_{g}(t+\tau ){\rm{d}}t}}\limits_{\equiv \Delta S(p+{A}_{p}(t),{t}_{2},{t}_{1};{A}_{g}(t+\tau ))}\end{array}$$

Here the square term of *A*_g_(*t* + *τ*) is ignored, as its contribution is negligible compared with the other terms. Equation (10) implies that the gate field introduces an extra phase of $\Delta S(p+{A}_{{\rm{p}}}(t),{t}_{2},{t}_{1}\,;{A}_{{\rm{g}}}(t+\tau ))$ to the gate-free case. As a result, equation (9) can be rewritten as:

11 $${\psi }_{{\rm{r}}}^{({\rm{g}})}({t}_{{\rm{r}}},\tau )\,\approx {\int }_{-\infty }^{{t}_{{\rm{r}}}}{\rm{d}}{t}^{{\prime} }\int {\rm{d}}{p}^{{\prime} }g({p}^{{\prime} }+{A}_{{\rm{p}}}({t}_{{\rm{r}}})){E}_{{\rm{p}}}({t}^{{\prime} })d({p}^{{\prime} }+{A}_{{\rm{p}}}({t}^{{\prime} }))\times \exp \left[-{\rm{i}}S({p}^{{\prime} },{t}_{{\rm{r}}},{t}^{{\prime} }{\rm{;}}{A}_{{\rm{p}}}(t))-{\rm{i}}\frac{{p}^{2}}{2}({t}_{{\rm{r}}}-{t}^{{\prime} })+{\rm{i}}\phi {t}^{{\prime} }\right]\exp \left[-{\rm{i}}\Delta S({p}^{{\prime} }+{A}_{{\rm{p}}}(t),{t}_{{\rm{r}}},{t}^{{\prime} }{\rm{;}}{A}_{{\rm{g}}}(t+\tau ))\right]$$

Note that if the last phase term e^−iΔ*S*^ was missing, the expression would be identical to equation (7) and, thus, the gate-free recollision wave function *ψ*_r_(*t*_r_). Hence it would obviously be convenient to remove the e^−iΔ*S*^ term from the integrals, as this would enable to express the recolliding electron wave packet ${\psi }_{{\rm{r}}}^{({\rm{g}})}({t}_{{\rm{r}}},\tau )$ in equation (8) through the unperturbed counterpart *ψ*_r_(*t*_r_) and an extra phase. To proceed further with this idea, we consider two approximations.

First, following the famous saddle-point approximation, the dominant contribution in the integration over the canonical momenta *p*′ is provided by the kinetic momentum *p*′ + *A*_p_(*t*) that equals the kinetic momentum *p*_r_ of the recolliding electron at the surface. Hence the kinetic momentum term in Δ*S* may be approximated as $\Delta S({p}^{{\prime} }{+A}_{{\rm{p}}}(t),{t}_{{\rm{r}}},{t}^{{\prime} }{\rm{;}}{A}_{{\rm{g}}}(t+\tau ))\approx \Delta S({p}_{{\rm{r}}},{t}_{{\rm{r}}},{t}^{{\prime} }{\rm{;}}{A}_{{\rm{g}}}(t+\tau ))$.

Second, because the exponent term of the extra phase e^−iΔ*S*^ is oscillating slowly compared with e^−i*S*^ in the time integration over *t*′ in equation (11), the extra phase Δ*S* can be approximated by a time average $\overline{\Delta S}$ within a time window Δ*t*:

12 $$\overline{\Delta S}=\frac{1}{\left|\Delta t\right|}{\int }_{{t}_{{\rm{r}}}-\Delta t}^{{t}_{{\rm{r}}}}\Delta S({p}_{{\rm{r}}},{t}_{{\rm{r}}},{t}^{{\prime} },{A}_{{\rm{g}}}(t+\tau )){\rm{d}}{t}^{{\prime} }$$

As our main goal is to reconstruct attosecond electron wave packets that contribute to the spectral cutoff, we choose Δ*t* as the time interval between ionization and recollision of the classical backscattering trajectory that results in the highest final kinetic energy. To evaluate the averaged phase $\overline{\Delta S}$ and thereby simplify the analytical form of equation (12), Δ*S* can be expressed as:

13 $$\begin{array}{c}\Delta S({p}_{{\rm{r}}},{t}_{{\rm{r}}},{t}^{{\prime} },{A}_{{\rm{g}}}(t+\tau ))={\int }_{{t}^{{\prime} }}^{{t}_{{\rm{r}}}}{p}_{{\rm{r}}}{A}_{{\rm{g}}}(t+\tau ){\rm{d}}t=-{\int }_{{t}_{{\rm{r}}}}^{{\rm{\infty }}}{p}_{{\rm{r}}}{A}_{{\rm{g}}}(t+\tau ){\rm{d}}t\\ \,\,\,\,\,+{\int }_{{t}^{{\prime} }}^{{\rm{\infty }}}{p}_{{\rm{r}}}{A}_{{\rm{g}}}(t+\tau ){\rm{d}}t\end{array}$$

By inserting equation (13) into equation (12), the effective (averaged) phase variation $\overline{\Delta S}\,$ can now be evaluated:

14 $$\begin{array}{l}\overline{\Delta S}=-{\int }_{{t}_{r}}^{\infty }{p}_{r}{A}_{g}(t+\tau ){\rm{d}}t+{\int }_{{t}_{r}}^{\infty }{p}_{r}\mathop{\underbrace{\left[\frac{1}{\Delta t}{\int }_{-\Delta t}^{0}{A}_{g}(t+t{\prime} +\tau )\,{\rm{d}}t{\prime} \right]}}\limits_{\equiv {\bar{A}}_{g}^{(b)}(t+\tau )}{\rm{d}}t\\ \,=-{\int }_{{t}_{r}}^{\infty }{p}_{r}[{A}_{g}(t+\tau )-{\bar{A}}_{g}^{(b)}(t+\tau )]\,{\rm{d}}t\\ \,=-\Delta {\rm{S}}({p}_{r},\,\infty ,{{\rm{t}}}_{{\rm{r}}};{A}_{g}(t+\tau )-{\bar{A}}_{g}^{(b)}(t+\tau ))\end{array}$$

in which ${\bar{A}}_{{\rm{g}}}^{({\rm{b}})}(t)$ is defined as:

15 $${\bar{A}}_{{\rm{g}}}^{({\rm{b}})}(t)=\frac{1}{\Delta t}{\int }_{-\Delta t}^{0}{A}_{{\rm{g}}}(t+{t}^{{\prime} }){\rm{d}}{t}^{{\prime} }$$

Using the above-described approximations now enables to pull the extra phase term out of the integrations in equation (11) and, considering the sign flip $\overline{\Delta S}\to -\overline{\Delta S}$ at the backscattering instance, the perturbed recolliding electron wave packet can be expressed through the gate-free wave packet and the extra averaged phase term ${\psi }_{{\rm{r}}}^{({\rm{g}})}({t}_{{\rm{r}}},\tau )\approx {\psi }_{{\rm{r}}}({t}_{{\rm{r}}}){{\rm{e}}}^{{\rm{i}}\overline{\Delta S}}$ in equation (8).

We now move on to discuss how the electron wave packet can be described at the end of the interaction in the presence of the pump and gate fields (equation (8)). Taking the results of equations (11)–(14) and restoring *p*_r_ with the kinetic momentum *p* + *A*_p_(*t*), equation (8) can be rewritten as:

16 $${\widetilde{\psi }}_{{\rm{t}}}(p,\tau )\propto {\rm{i}}{\int }_{-\infty }^{\infty }{\psi }_{{\rm{r}}}({t}_{{\rm{r}}})\exp \left[{\rm{i}}\frac{{p}^{2}}{2}{t}_{r}\right]\exp \left[-{\rm{i}}S(p,\infty ,{t}_{{\rm{r}}}{\rm{;}}{A}_{{\rm{p}}}(t)+{A}_{{\rm{g}}}(t+\tau ))\right]\times \exp [-{\rm{i}}\Delta S(p+{A}_{{\rm{p}}}(t),\infty ,{t}_{{\rm{r}}}{\rm{;}}{A}_{{\rm{g}}}\left(t+\tau \right)-{\bar{A}}_{{\rm{g}}}^{({\rm{b}})}(t+\tau ))]{\rm{d}}{t}_{{\rm{r}}}$$

Because the integration ranges for *S* and Δ*S* are identical (from *t*_r_ to ∞), the two phases can be merged into a single equation (*S*′ = *S* + Δ*S*),

17 $$\begin{array}{c}S{\prime} (p,\infty ,{t}_{r},\tau )={\int }_{{t}_{r}}^{\infty }[\frac{1}{2}{[p+{A}_{p}(t)+{A}_{g}(t+\tau )]}^{2}-\frac{1}{2}{p}^{2}]{\rm{d}}t\\ \,\,\,+\,{\int }_{{t}_{r}}^{\infty }(p+{A}_{p}(t))({A}_{g}(t+\tau )-{\bar{A}}_{g}^{(b)}(t+\tau )){\rm{d}}t\\ \,\,\,\approx \,{\int }_{{t}_{r}}^{\infty }[\frac{1}{2}{[p+{A}_{p}(t)+\mathop{\underbrace{2{A}_{g}(t+\tau )-{\bar{A}}_{g}^{(b)}(t+\tau )}}\limits_{\equiv {A}_{HAS}(t+\tau )}]}^{2}\\ \,\,\,-\,\frac{1}{2}{p}^{2}]{\rm{d}}t=S(p,\infty ,{t}_{r}\,;{A}_{p}(t)+{A}_{HAS}(t+\tau ))\end{array}$$

in which *A*_HAS_(*t*) is hereafter referred to as the effective HAS vector potential and reads:

18 $${A}_{{\rm{HAS}}}(t)=2{A}_{{\rm{g}}}(t)-{\bar{A}}_{{\rm{g}}}^{({\rm{b}})}(t)$$

This expression of the effective HAS vector potential is compatible with the classical momentum accumulation during the excursion from the ionization to the detection, $\Delta p=-\,{\rm{e}}\left[2A({t}_{{\rm{r}}})-A({t}_{{\rm{r}}}-\Delta t)\right]$, under the rescattering condition, ${\int }_{{t}_{{\rm{r}}}-\Delta t}^{{t}_{{\rm{r}}}}{A}_{{\rm{g}}}(t){\rm{d}}t=\Delta t{A}_{{\rm{g}}}({t}_{{\rm{r}}}-\Delta t)$(refs. ^11,36,63^). Using equation (17), the terminal electron amplitude ${\widetilde{\psi }}_{{\rm{t}}}(p,\tau )$ can be expressed as:

19 $$\begin{array}{c}{\widetilde{\psi }}_{{\rm{t}}}(p,\tau )\propto {\rm{i}}{\int }_{-\infty }^{\infty }{\psi }_{{\rm{r}}}({t}_{{\rm{r}}})\,\exp \,\left[{\rm{i}}\frac{{p}^{2}}{2}{t}_{{\rm{r}}}\right]\,\exp [-{\rm{i}}S(p,\infty ,{t}_{{\rm{r}}}{\rm{;}}{A}_{{\rm{p}}}(t)\\ \,\,+{A}_{{\rm{HAS}}}(t+\tau ))]{\rm{d}}{t}_{{\rm{r}}}\end{array}$$

Equation (19) implies that the gate also contributes to the terminal momentum of the electron wave packet by *A*_HAS_(*t*_r_ + *τ*), which depends on the time delay *τ*. As described in equation (5), the momentum contribution *A*_p_(*t*) of the pump field is already incorporated in the gate-free terminal electron spectral amplitude ${\widetilde{\psi }}_{{\rm{t}}}(p)$, whose intensity is directly accessible in experiments. Therefore it is convenient for the analysis of HAS data to express equation (19) with the terminal form of ${\widetilde{\psi }}_{{\rm{t}}}(p).$ We decompose the phase in equation (19) by means of $S(p,{t}_{2},{t}_{1}\,;{A}_{{\rm{p}}}(t)+{A}_{{\rm{g}}}(t+\tau ))\approx S(p,{t}_{2},{t}_{1}\,;{A}_{{\rm{p}}}(t))+\Delta S(p+{A}_{{\rm{p}}}(t),{t}_{2},{t}_{1}\,;{A}_{{\rm{HAS}}}(t+\tau ))$ and rewrite equation (19) as:

20 $${\widetilde{\psi }}_{{\rm{t}}}(p,\tau )\propto {\rm{i}}{\int }_{-\infty }^{\infty }{\psi }_{{\rm{r}}}({t}_{{\rm{r}}})\exp \left[{\rm{i}}\frac{{p}^{2}}{2}{t}_{{\rm{r}}}\right]\exp \left[-{\rm{i}}S(p,\infty ,{t}_{{\rm{r}}}{\rm{;}}{A}_{{\rm{p}}}(t))\right]\times \exp \left[-{\rm{i}}\Delta S(p+{A}_{{\rm{p}}}(t),\infty ,{t}_{{\rm{r}}}{\rm{;}}{A}_{{\rm{HAS}}}(t+\tau ))\right]{\rm{d}}{t}_{{\rm{r}}}$$

In analogy to equation (11), if the e^−iΔ*S*^ term vanishes, the above equation is identical to equation (5), which links the recolliding electron wave packet *ψ*_r_(*t*) to the terminal spectral amplitude ${\widetilde{\psi }}_{{\rm{t}}}(p)$. Here, similar to the Fourier representation of the recollision wave packet, we define the Fourier pair of the terminal electron wave packet as:

21 $$\begin{array}{c}{\widetilde{\psi }}_{{\rm{t}}}(p)\equiv \,{\int }_{-\infty }^{\infty }{\rm{d}}t\,{\psi }_{{\rm{t}}}(t)\,\exp \left[i\frac{{p}^{2}}{2}t\right],\\ \,{\psi }_{{\rm{t}}}(t)\equiv \,{\int }_{-\infty }^{\infty }p{\rm{d}}p\,{\widetilde{\psi }}_{{\rm{t}}}(p)\,\exp \left[-i\frac{{p}^{2}}{2}t\right]\end{array}$$

Note that the terminal electron wave packet *ψ*_t_(*t*) is an auxiliary electron wave packet that contains time–structure information of the recolliding electron wave packet *ψ*_r_(*t*) at the recollision surface with the momenta translated by the Volkov propagation with the exponent $\exp \left[-{\rm{i}}S(p,\infty ,{t}_{{\rm{r}}}{\rm{;}}{A}_{{\rm{p}}}(t))\right]$ (see equations (1) and (5)), but without the phase from space propagation to the detection. Using the terminal electron wave packet *ψ*_t_(*t*) (equation (21)), the terminal electron spectral amplitude (equation (20)) can be further simplified as:

22 $${\widetilde{\psi }}_{{\rm{t}}}(p,\tau )\propto {\rm{i}}{\int }_{-\infty }^{\infty }{\psi }_{{\rm{t}}}({t}_{{\rm{r}}})\exp \left[{\rm{i}}\frac{{p}^{2}}{2}{t}_{{\rm{r}}}\right]\exp \left[-{\rm{i}}S(p,\infty ,{t}_{{\rm{r}}}{\rm{;}}{A}_{{\rm{HAS}}}(t+\tau ))\right]{\rm{d}}{t}_{{\rm{r}}}$$

under the condition that the variation of the vector potential is weak during the time window of the recollision. The HAS spectrogram equation then reads:

23 $$I(p,\tau )={\left|{\widetilde{\psi }}_{{\rm{t}}}(p,\tau )\right|}^{2}\propto {\left|{\int }_{-\infty }^{\infty }{\psi }_{{\rm{t}}}({t}_{{\rm{r}}})\exp \left[{\rm{i}}\frac{{p}^{2}}{2}{t}_{{\rm{r}}}\right]\exp \left[-{\rm{i}}S(p,\infty ,{t}_{{\rm{r}}}{\rm{;}}{A}_{{\rm{HAS}}}(t+\tau ))\right]{\rm{d}}{t}_{{\rm{r}}}\right|}^{2}$$

Equation (23) describes a spectrogram whose reconstruction allows access to the final electron wave packet *ψ*_t_(*t*) and, correspondingly, ${\widetilde{\psi }}_{{\rm{t}}}(p)$ as well as *A*_HAS_(*t*).

#### The effective HAS vector potential *A*_HAS_(*t*)

An explicit relationship between the incident gate vector potential *A*_g_(*t*) and the effective HAS vector potential *A*_HAS_(*t*) can be best understood in the Fourier domain. Using the Fourier expansion, ${A}_{{\rm{g}}}(t)={\int }_{-{\rm{\infty }}}^{{\rm{\infty }}}{\rm{d}}\omega {\widetilde{A}}_{{\rm{g}}}(\omega ){{\rm{e}}}^{{\rm{i}}\omega t}$, the effective HAS vector potential can be expressed as,

24 $${A}_{{\rm{HAS}}}(t)=2{\int }_{-{\rm{\infty }}}^{{\rm{\infty }}}{\widetilde{A}}_{{\rm{g}}}(\omega ){{\rm{e}}}^{i\omega t}{\rm{d}}\omega -\frac{1}{\Delta t}{\int }_{-\Delta t}^{0}{\int }_{-{\rm{\infty }}}^{{\rm{\infty }}}{\widetilde{A}}_{{\rm{g}}}\left(\omega \right){e}^{i\omega (t+{t}^{{\prime} })}{\rm{d}}\omega {\rm{d}}t{\prime} ={\int }_{-{\rm{\infty }}}^{{\rm{\infty }}}{\widetilde{A}}_{{\rm{g}}}\left(\omega \right)\left[2-\frac{i}{\omega \Delta t}({e}^{-i\omega \Delta t}-1)\right]{e}^{i\omega t}{\rm{d}}\omega ={\int }_{-{\rm{\infty }}}^{{\rm{\infty }}}{\widetilde{A}}_{{\rm{g}}}\left(\omega \right)\widetilde{g}(\omega ){e}^{i\omega t}{\rm{d}}\omega $$

in which the newly introduced multiplier $\widetilde{g}(\omega )$ is defined as:

25 $$\widetilde{g}(\omega )=\left[2-\frac{{\rm{i}}}{\omega \Delta t}({{\rm{e}}}^{-{\rm{i}}\omega \Delta t}-1)\right]$$

As shown in equation (24), the Fourier components of the effective HAS vector potential ${\widetilde{A}}_{{\rm{HAS}}}(\omega )$ is related to those of the incident gate vector potential ${\widetilde{A}}_{{\rm{g}}}(\omega )$ by multiplication of $\widetilde{g}(\omega )$

26 $${\widetilde{A}}_{{\rm{HAS}}}(\omega )={\widetilde{A}}_{{\rm{g}}}(\omega )\widetilde{g}(\omega )$$

The gate multiplier $\widetilde{g}(\omega )$ is independent from ${\widetilde{A}}_{{\rm{g}}}(\omega )$. This allows the possibility of the complete characterization of *A*_g_(*t*) from *A*_HAS_(*t*) imprinted in a HAS spectrogram.

**Extended Data Fig. 7:**
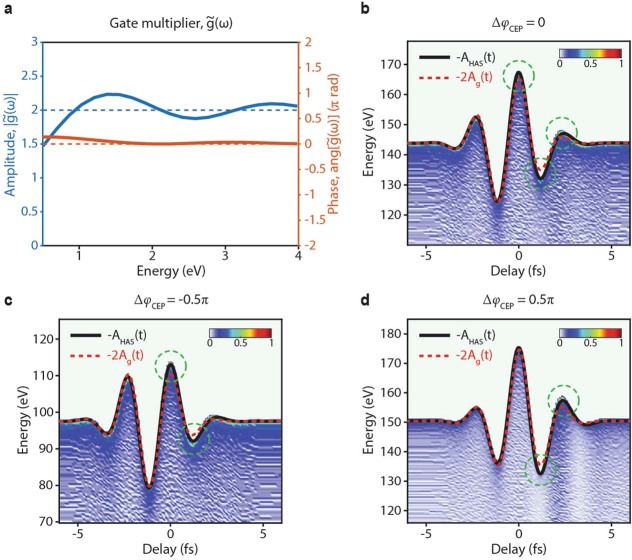
Incident and effective HAS gate vector potentials. **a**, Gate multiplier function $\widetilde{g}(\omega )$ as defined in the text. **b**–**d**, Semiclassically simulated HAS spectrograms at different carrier-envelope phase (CEP) settings of the driving pulse. The red dashed and black solid curves indicate waveforms of the incident gate vector potential *A*_g_(*t*) and effective HAS gate vector potential *A*_HAS_(*t*) calculated using equation (24), respectively. Source data

To better visualize the concept of *A*_HAS_(*t*) and to verify the validity of the assumptions used in the above derivation, a semiclassical simulation of a HAS spectrogram was performed using single-cycle pulses. The photoelectron spectrum cutoff energy variation evaluated by the HAS spectrogram is compared with the effective HAS vector potential *A*_HAS_(*t*) calculated using equation (24) (Extended Data Fig. 7). The multiplier $\widetilde{g}(\omega )$ depends on the excursion time Δ*t* between ionization and the backscattering event of the highest-energy electron. On the basis of the well-established recollision model, 0.685 times the central excursion period^64,65^ (central period of $E(\omega )/\left.{\omega }^{2}\right)$), which corresponds to about 0.85*T*_L_, was used for Δ*t* to evaluate $\widetilde{g}(\omega )$. Here *T*_L_ is the centroid period of the laser pulse. Extended Data Fig. 7b–d shows that the cutoff energy variation in a HAS spectrogram closely follows *A*_HAS_(*t*) (black curve), as calculated by the unmodified vector potential of the incident pulse (dashed red curve), regardless of the carrier-envelope phase.

### Retrieval of the vector potential *A*_g_(*t*) from a HAS spectrogram

**Extended Data Fig. 8:**
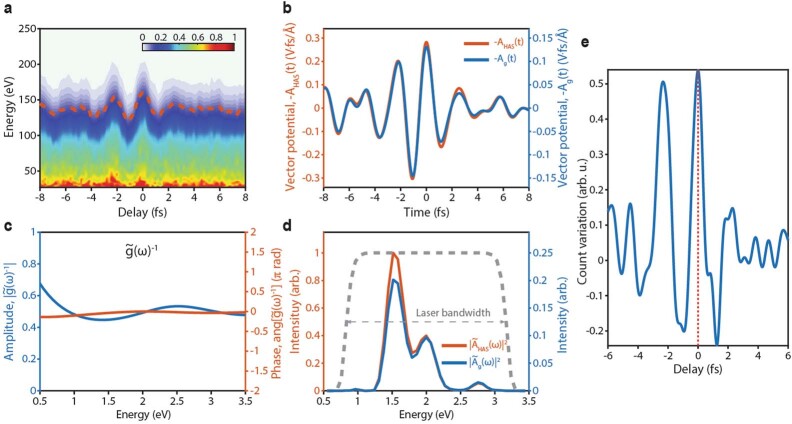
Retrieval of the vector potential of the incident gate field. **a**, Measured HAS spectrogram (Fig. 3a). **b**, *A*_HAS_(*t*) (red) directly extracted by tracing the cutoff energy variation in the spectrogram and the retrieved incident vector potential *A*_g_(*t*) (blue). **c**, Amplitude (blue) and phase (red) of the inverse of the multiplier $\widetilde{g}{\left(\omega \right)}^{-1}$. **d**, Spectral intensity of *A*_HAS_(*t*) (red) and incident *A*_g_(*t*) (blue) vector potentials. **e**, Identification of timing between gate and pump pulses in a HAS spectrogram. The variation of electron yields is evaluated by the spectral integration of the HAS spectrogram (blue curve) for each delay. The absolute zero delay between gate and pump pulses is identified as the point at which the yield variation is maximized (dashed red line). Source data

The above discussion suggests that, by tracing the variation of the cutoff energy in a HAS spectrogram, we can obtain *A*_HAS_(*t*) (red curves in Extended Data Fig. 8a,b). Therefore access to the Fourier components of the effective HAS vector potential allows the characterization of the vector potential of the incident gate: ${\widetilde{A}}_{{\rm{g}}}(\omega )={\widetilde{g}}^{-1}(\omega ){\widetilde{A}}_{{\rm{HAS}}}(\omega )$ (Extended Data Fig. 8c,d). The retrieved incident vector potential *A*_g_(*t*) is shown in blue in Extended Data Fig. 8b.

#### Identification of the absolute zero delay in a HAS spectrogram

Identification of the zero delay between pump and gate pulses in a HAS spectrogram can be obtained with various methods. Here we opted for a method that allows the absolute delay to be derived directly from the HAS spectrogram. Even though the difference between the intensities of pump and gate pulses is more than two orders of magnitude, discernible modulations (approximately 5–10%) of the spectral amplitude of the spectrogram remain. In a HAS spectrogram, the total photoelectron yield variation can be evaluated by spectral integration at each delay point (Extended Data Fig. 8e). The absolute zero-delay point can be found as the delay point at which the yield is maximally varied (vertical dashed line in Extended Data Fig. 8e).

#### Benchmarking HAS through EUV attosecond streaking

EUV attosecond streaking provides access to the detailed field waveform of a pulse^33–35^. Because this technique of field characterization is integrated in our experimental setup, it allows us to benchmark HAS as a field-characterization method.

**Extended Data Fig. 9:**
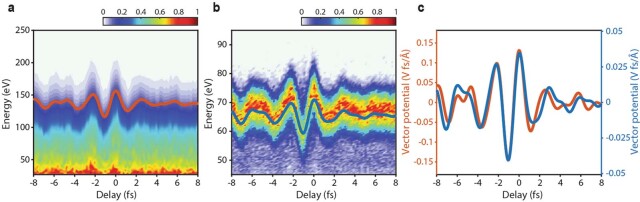
Benchmarking of HAS through EUV attosecond streaking. HAS (**a**) and EUV attosecond streaking (**b**) spectrograms under identical driving-pulse field. **c**, Comparison of the vector potential of the gate pulse evaluated from HAS (red) and EUV attosecond streaking (blue) techniques. Source data

Extended Data Fig. 9a,b shows the HAS and EUV attosecond streaking measurements, respectively. The vector potential waveform of the incident gate pulse retrieved from the cutoff analysis in HAS (red curve in Extended Data Fig. 9c) and that from EUV attosecond streaking (blue curve in Extended Data Fig. 9c) show excellent agreement, as verified by the degree of similarity of about 0.95 (ref. ^43^), and support the notion that the gate pulse indeed acts as a phase gate.

### The gate pulse as a phase gate

The compact description of HAS as a spectrogram implied by equations (2) and (23) assumes that the weak replica of the pump field acts as a nearly pure phase gate on the electrons released by the pump. In other words, it can modify the momentum of electrons released by the pump field but does not greatly influence the process of electron ionization. Yet, unless the ionization nonlinearities are well understood (for instance, in atoms), a theoretical estimate of the required ratio between pump and gate pulses for attaining a sufficiently pure phase gate requires experimental validation.

**Extended Data Fig. 10:**
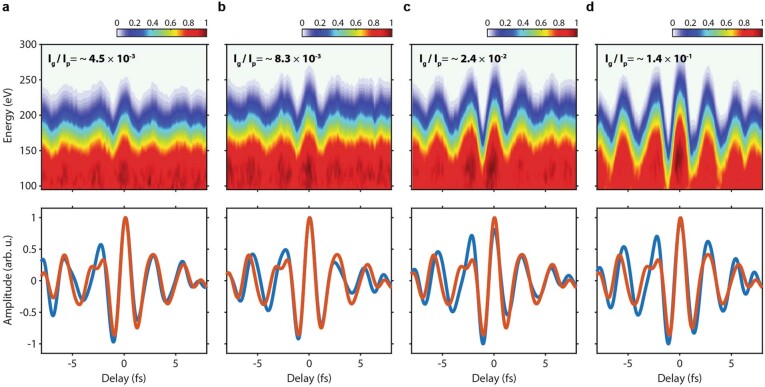
Benchmarking HAS. Top panels, HAS spectrograms recorded at different gate/pump intensity ratios, *η* ≈ 4.5 × 10^−3^ (**a**), 8.3 × 10^−3^ (**b**), 2.4 × 10^−3^ (**c**) and 1.4 × 10^−3^ (**d**). Bottom panels, comparison of the vector potential waveform characterized by EUV attosecond streaking (red curve) and HAS (blue curve) for each gate/pump intensity ratio. Source data

To identify safe limits within which the above condition is met, we performed HAS measurements under different gate strengths and compared the vector potential waveforms extracted from HAS to those characterized by EUV attosecond streaking. As shown in Extended Data Fig. 10a,b, the vector potential waveforms from two techniques achieve best agreement at low gate/pump intensity ratio (*η* < 10^−2^). At higher intensity ratios, we observe a gradually increasing disagreement between the reconstructed waveforms with the two methods (Extended Data Fig. 10c,d), implying that the gate pulse no longer serves as a weak perturbation. These measurements suggest that, for the studied system, HAS measurements require a gate pulse whose intensity is about 10^−2^ lower than the pump intensity.

### HAS reconstruction methodology

At the first stage of the reconstruction of the HAS spectrogram, the terminal electron wave packet *ψ*_t_(*t*) is retrieved, as its spectral intensity ${\left|{\widetilde{\psi }}_{{\rm{t}}}(p)\right|}^{2}$ can be directly obtained by a gate-free photoelectron spectrum. Therefore the reconstruction problem is reduced to retrieval of the spectral phase.

**Extended Data Fig. 11:**
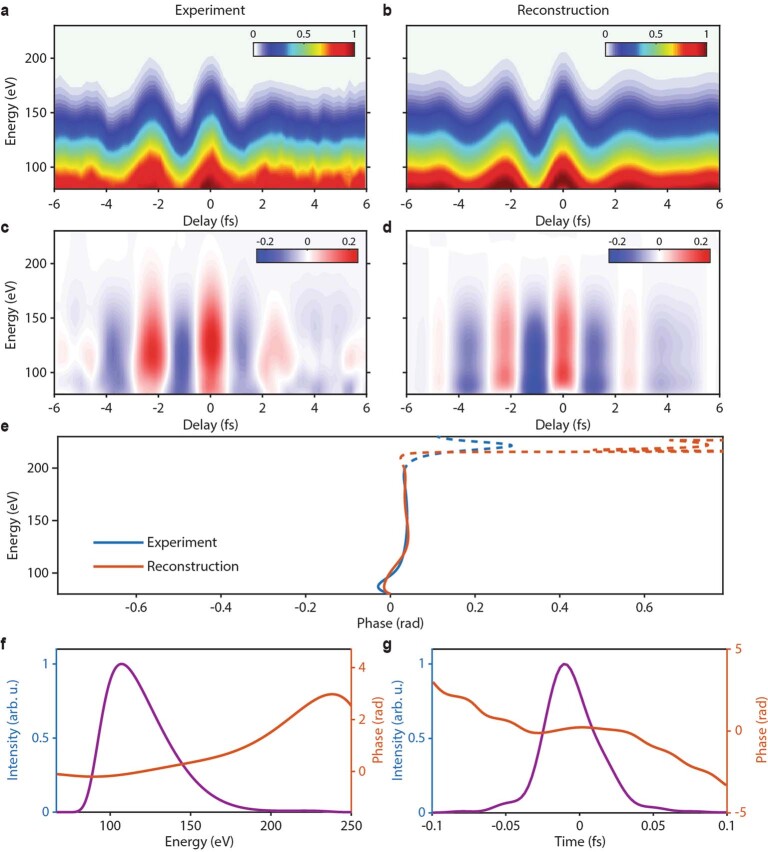
Retrieval of the terminal electron wave packet. Measured (**a**) and reconstructed (**b**) HAS spectrograms in the energy range from 80 to 230 eV. Differential maps of measured (**c**) and reconstructed (**d**) spectrograms. **e**, The phase of the intensity shift in measured (blue) and reconstructed (red) spectrograms. Retrieved terminal electron wave packet in spectral (**f**) and temporal (**g**) domains. The purple and red curves denote the intensity and phase, respectively. Source data

As motivated in the main text, we isolated a spectral area of interest (AOI) from 80 to 230 eV (Extended Data Fig. 11a,b). With this region of interest, the terminal wave packet can be expressed as:

27 $${\psi }_{{\rm{t}}}^{({\rm{AOI}})}(t)={\int }_{-{\rm{\infty }}}^{{\rm{\infty }}}\left|{\widetilde{\psi }}_{{\rm{t}}}^{({\rm{AOI}})}(\omega )\right|{{\rm{e}}}^{-{\rm{i}}{\varphi }\left({\rm{\omega }}\right)}{{\rm{e}}}^{{\rm{i}}\omega t}{\rm{d}}\omega $$

Here *φ*(*ω*) is the spectral phase of the electron wave packet modelled as a polynomial series up to the sixth order,

28 $$\varphi (\omega )=\mathop{\sum }\limits_{n}^{N=6}{D}_{n}{(\omega -{\omega }_{{\rm{c}}})}^{n}$$

in which *D*_*n*_ and *ω*_c_ are the *n*th order dispersion and central frequency, respectively. The reconstruction is based on a least-squares algorithm written in MATLAB, which aims at the total minimization of the difference among the experimental (Fig. 4a and Extended Data Fig. 11a) and reconstructed spectrogram (Fig. 4b and Extended Data Fig. 11b). To further increase the fidelity of the reconstruction, we also simultaneously fit the differential map *D*(*E*, *τ*) of a HAS spectrogram *I*(*E*, *τ*), which is defined as:

29 $$D(E,\tau )=\int \frac{\partial I(E,\tau )}{\partial \tau }{\rm{d}}\tau $$

The differential map is useful because it can eliminate the unmodulated intensity along the delay axis and allows the retrieval algorithm to reconstruct fine details of the experimental trace (Extended Data Fig. 11c–e). As an initial guess for the phase, zero phase was used. The retrieval of the terminal electron wave packet is shown in Extended Data Fig. 11f,g.

In a next stage of the reconstruction, the recolliding electron pulse, which is the key quantity in this work, is evaluated by the inverse Volkov propagation of the retrieved terminal electron wave packet as:

30 $${\psi }_{{\rm{r}}}^{({\rm{AOI}})}(t)={\int }_{-\infty }^{\infty }{\rm{d}}p\,{\widetilde{\psi }}_{{\rm{t}}}^{({\rm{AOI}})}(p)\exp \,\left[-{\rm{i}}\frac{{p}^{2}}{2}t\right]\,\exp \left[{\rm{i}}S(p,\infty ,t{\rm{;}}{A}_{{\rm{p}}}(t))\right]$$

The Volkov basis is reconstructed by precisely measuring the pump-field waveform and its timing with respect to the emission. The retrieved recolliding electron pulse is shown in Fig. 4.

## Online content

Any methods, additional references, Nature Portfolio reporting summaries, source data, extended data, supplementary information, acknowledgements, peer review information; details of author contributions and competing interests; and statements of data and code availability are available at 10.1038/s41586-022-05577-1.

## Data Availability

The data supporting the conclusions in the paper are available from the corresponding authors on reasonable request. Source data are provided with this paper.

## Source data

Source Data Fig. 1
Source Data Fig. 2
Source Data Fig. 3
Source Data Fig. 4
Source Data Extended Data Fig. 3
Source Data Extended Data Fig. 4
Source Data Extended Data Fig. 5
Source Data Extended Data Fig. 6
Source Data Extended Data Fig. 7
Source Data Extended Data Fig. 8
Source Data Extended Data Fig. 9
Source Data Extended Data Fig. 10
Source Data Extended Data Fig. 11
